# Phase separation: a new window in RALF signaling

**DOI:** 10.3389/fpls.2024.1409770

**Published:** 2024-06-27

**Authors:** Zilin Zhang, Huiming Deng, Songping Hu, Huibin Han

**Affiliations:** Research Center of Plant Functional Genes and Tissue Culture Technology, College of Bioscience and Bioengineering, Jiangxi Agricultural University, Nanchang, China

**Keywords:** RALF peptide, FER, LLG1, LRX, phase separation, pectin, GRP7

## 1 Introduction

The RAPID ALKALINIZATION FACTOR (RALF) peptides belong to the cysteine-rich small peptide family, they play essential roles in plant growth and development and biotic and abiotic stress responses (Blackburn et al., 2020; Zhang et al., 2020; Somoza et al., 2021; Cheung, 2024). RALF proteins contain an N-terminal signal peptide, a conserved dibasic site (RR motif) that is essential for proper maturation and release of RALFs, a conserved YISY motif that is crucial for GLYCOSYLPHOSPHATIDYLINOSITOL-ANCHORED PROTEIN (GPI-AP) LORELEI (LRE) and (LRE)-LIKE-GPI-AP1 (LLG) receptors binding but is not universally presented in all RALF members, tyrosine motifs for the cell-wall-anchored protein LEUCINE-RICH-REPEAT EXTENSIN (LRX) binding, and four cysteines at the C-terminus that have been shown to be crucial for disulfide bridges formation and protein conformation (Blackburn et al., 2020). The disulfide bridges lead to the formation of two loops (C21–29 and C41–47) potentially influencing the conformation and bioactivity of RALF peptides (Frederick et al., 2019). The *Catharanthus roseus* RLK1-LIKE (CrRLK1L) receptor kinase FERONIA (FER) (Haruta et al., 2014; Stegmann et al., 2017), the LLG co-receptors (Li et al., 2015), and LRXs (Draeger et al., 2015; Mecchia et al., 2017; Zhao et al., 2018; Herger et al., 2019) can recognize RALF ligands. They can (de)activate multiple downstream signaling cascades, thereby controlling a wide range of plant developmental and adaptive processes (Zhu et al., 2021; Cheung, 2024). The mechanisms underlying RALF signaling activation through LRX, LLG1, and FER proteins, however, remain largely unknown.

**Figure 1:**
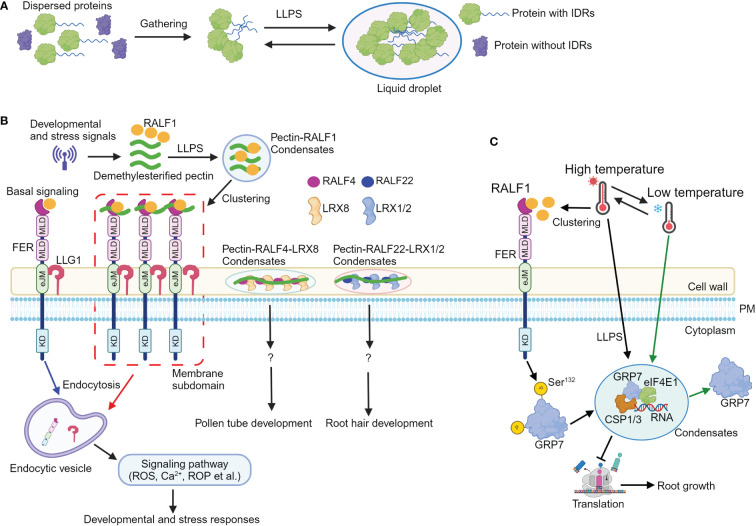
Liquid–liquid phase separation mediates RALF-FERONIA signaling in plant development and stress responses. **(A)** A simple schematic diagram illustrating the process of proteins undergoing liquid–liquid phase separation (LLPS). Proteins containing intrinsically disordered regions (IDRs) first gather together, and then, they undergo LLPS to form liquid droplet structure. **(B)** Upon sensing developmental or stress cues, the levels of apoplastic demethylesterified pectic fragments and RALF1 are increased. Subsequently, RALF1 and pectin undergo LLPS to generate RALF1-pectin condensates. RALF1-pectin condensates incorporate FER and LLG1 receptors; the RALF-pectin-FER-LLG1 condensates then localize to the microdomain at plasma membrane, thus activating endocytosis of FER and LLG1 receptors, ultimately (de)activating multiple signals such as Ca^2+^, ROS, and ROP to coordinate plant development and stress responses. Additionally, RALF4 and RALF22 bind to pectin, forming pectin-RALF4-LRX8 and pectin-RALF22-LRX1/2 condensates to regulate pollen tube and root growth, respectively. **(C)** Elevated temperatures enhance the levels and clustering of RALF1 peptide; FERONIA then perceives RALF1 ligand to phosphorylate itself and GRP7 at Ser^132^ site, which contributes to GRP7 LLPS. The GRP7 condensates incorporate components including CSP1/3, eIF4E1, and RNA to modulate translation and root growth, thus endowing seedlings with temperature resilience. Notably, low temperatures reverse GRP7 phase separation. eJM, extracellular juxta-membrane domain; KD, kinase domain; MLD, malectin-like domain; PM, plasma membrane; ROS, reactive oxygen species; ROP, Rho-of-Plant. ?, indicates unknown signaling component.

In recent years, advancements in subcellular organization analysis have significantly increased the number of known membrane-less compartments in cells. They are widely distributed in the nucleus, the cytoplasm, and in membranes, and are grouped as biomolecular condensates (Emenecker et al., 2020; 2021). Liquid–liquid phase separation (LLPS) is a major driving force for the formation of biomolecular condensates. LLPS triggers the condensation of scattered macromolecules such as proteins or nucleic acids into a more condensed phase to form a liquid droplet structure in a certain cellular environment (**Figure 1A**; Hyman et al., 2014). Intrinsically disordered proteins (IDPs) could recruit diverse molecules through specific and multivalent interactions, concentrating them into biomolecular condensates via LLPS, enabling spatiotemporal regulation of a variety of cellular activities. Proteins undergoing phase separation typically contain intrinsically disordered regions (IDRs) or low complexity domains (LCDs), which are essential for biomolecular condensate formation (Das et al., 2015; Alberti and Hyman, 2021). A myriad of plant biological processes are regulated by LLPS (Liu et al., 2024a), as indicated by a range of evidence, including photomorphogenesis (Mo et al., 2022), flowering (Huang et al., 2021), immune responses (Wang et al., 2023), auxin and ethylene responses (Powers et al., 2019; Lu et al., 2022), senescence (Cheng et al., 2022), seed germination (Dorone et al., 2021), drought response (Wang et al., 2022), and temperature response (Tong et al., 2022). Recent reports demonstrate the formation of pectin-RALF-LRX (Moussu et al., 2023; Schoenaers et al., 2024) and pectin-RALF-FER-LLG1 condensates (Liu et al., 2024b) to activate RALF signaling pathways. Furthermore, LLPS drives the formation of GRP7-eIF4E1-RNA-CSP1/3 condensates in a RALF1-FER module-dependent manner to regulate temperature fluctuations (Xu et al., 2024). These novel findings add a novel window for RALF signaling pathway.

## 2 The pectin-RALF-LRX and pectin-RALF-LLG1-FER condensates activate RALF signaling in plant development and stress responses

The plant cell wall is central to plant growth and development (Cosgrove, 2024). It contains cellulose microfibrils and matrix polymers, which include polyanionic pectin. Pectin is generally classified into three major types: homogalacturonan (HG), rhamnogalacturonan I (RG-I), and RG-II (Atmodjo et al., 2013). Accumulated evidence shows that RALF peptide can bind to demethylesterified homogalacturonan *in vitro* [OG_DP6–15_, oligogalacturonide (OG) polymerization (DP) between 6 and 15, here after referred as deHG] (Moussu et al., 2023; Schoenaers et al., 2024). RALF4 and RAFL22 form complex with LRX8 and LRX1/2, respectively (Moussu et al., 2023; Schoenaers et al., 2024). The RALF4-LRX8 and RALF22-LRX1/2 complex then bind to deHG to form pectin-RALF4-LRX8 and pectin-RALF22-LRX1/2 condensates, thus creating a reticulate pattern and circumferential rings to regulate cell wall integrity and growth in pollen tube and root hairs, respectively (Moussu et al., 2023; Schoenaers et al., 2024). Notably, the binding ability of RALF4 and RALF22 to negatively charged epitopes within deHG and the formation of pectin-RALF-LRX condensates are disrupted when the positively charged arginines are replaced by neutral alanine residues, indicating the charge dependency of pectin-RALF-LRX condensates formation (Moussu et al., 2023; Schoenaers et al., 2024). This disruption of pectin-RALF-LRX condensates leads to defective RALF peptide-mediated pollen tube and root hair growth, suggesting the critical roles of pectin-RALF-LRX condensates in RALF signaling activation (**Figure 1B**; Moussu et al., 2023; Schoenaers et al., 2024).

Furthermore, the synthetic RALF1 peptide triggers endocytosis and clustering of FER and LLG1 receptors through the YISY binding region (Xiao et al., 2019; Liu et al., 2024b), suggesting that RALF1 concentrates FER and LLG1 into membrane subdomains and potentially activates the FER-LLG1 complex. Due to its interactions with cell wall matrix molecules such as LRX proteins, RALF1 may remain extracellular and is unable to enter the cells through endocytosis. As RALF4, RALF22, and FER bind to pectin (Feng et al., 2018; Xiao et al., 2019; Duan et al., 2020; Lin et al., 2022; Tang et al., 2022; Moussu et al., 2023; Schoenaers et al., 2024), therefore, it is likely that RALF1 can also bind cell wall pectin. Indeed, RALF1 interacts with variant sizes of deHG *in vitro* (Liu et al., 2024b). RALF1 is predicted to be largely disordered (Liu et al., 2024b), and pectin can undergo phase transition (Haas et al., 2021). Hence, it is plausible that LLPS could drive the formation of pectin-RALF1 condensates (Das et al., 2015; Emenecker et al., 2020; Alberti and Hyman, 2021; 2021). Recombinant RALF1 alone and short deHG fragments do not form typical phase-separated condensate *in vitro*. However, when combining synthetic and recombinant RALF1 with deHG *in vitro*, they assemble into condensates. In addition, dynamic pectin-RALF1-FER-LLG1 condensates have also been observed.

Consistent with the observation of pectin-RALF1 phase separation, disrupting the normal pectin environment in seedlings (McKenna et al., 2019; Li et al., 2021) or inhibiting LLPS with 1,6-hexanediol inhibitor (Molliex et al., 2015) obliterates RALF1-triggered biological responses including endocytosis and FER and LLG1 receptor clustering, root growth inhibition, Ca^2+^, and ROS burst (Liu et al., 2024b). Importantly, mutations of arginine and tyrosine residues in the C-terminal region profoundly attenuate RALF1 ability to form condensates with pectin, highlighting the essential role of these residues in pectin-RALF1 phase separation (Liu et al., 2024b). In addition, environmental stresses such as high temperature and salinity elevate RALF and pectin levels to drive the formation of pectin-RALF1-FER-LLG1 condensates. The pectin-RALF1 condensates then recruit FER and LLG1 to activate RALF signaling pathways, ultimately triggering the rapid developmental and adaptive responses (**Figure 1B**; Liu et al., 2024b).

Taken together, developmental signals or stresses elevate the level of RALFs and short demethylesterified pectin fragments. The RALF peptides bind to demethylesterified pectin, leading to the formation of pectin-RALF1-FER-LLG1 and pectin-RALF-LRX condensates, respectively. These condensates then activate RALF signaling, ultimately coordinating downstream cellular responses and physiological outputs for an optimal plant development (**Figure 1B**).

## 3 RALF1-FER module facilitates GRP7 phase separation to adapt to fluctuating temperatures

Environmental temperature fluctuations profoundly limit plant growth and yield (González-García et al., 2023), and plants have evolved multiple strategies to cope with thermal fluctuations (Ding and Yang, 2022). FER is known to sense low temperature, thus controlling root hair growth (Kim et al., 2021; Pacheco et al., 2023). *GLYCINE-RICH RNA-BINDING PROTEIN7* (*GRP7*) encodes a protein containing an N-terminal RNA recognition motif and a C-terminal glycine-rich domain (Czolpinska and Rurek, 2018). It acts as an RNA chaperone that destabilizes the secondary structures of RNA molecules during cold response (Kim et al., 2007). Previous study unravels that FER interacts with GRP7 (Wang et al., 2020). Upon activation by RALF1 peptide, FER phosphorylates GRP7 to enhance its mRNA binding ability and fine-tune stress responses (Wang et al., 2020). However, the precise mechanism by which FER modulates GRP7 function in response to temperature fluctuations remains unclear.

*GRP7* transcription is regulated by cold and heat (Kilian et al., 2007; Kim et al., 2007), and the *grp7* mutant displays significantly shorter primary roots and lower survival rates under freezing (−20°C) or high temperature (42°C), respectively (Xu et al., 2024), suggesting the critical role of GRP7 in acclimation to temperature fluctuations. The presence of IDRs in the C-terminal glycine-rich region of GRP7 suggests its potential for phase separation (Das et al., 2015). In *GRP7::GRP7-GFP* seedlings, high temperature induces the formation of GRP7 stress granules (SGs), a transient cytoplasmic condensate composed of mRNAs that are halted in translation initiation and loaded into mRNA ribonucleoproteins (mRNPs) (Molliex et al., 2015; Cao et al., 2020; Xu et al., 2024). Notably, LLPS of GRP7 is reversed by low temperature. In addition, recombinant GRP7 proteins also form GRP7 condensates, while the truncated variant with the N-terminal of GRP7 does not undergo LLPS. These data suggest that GRP7 undergoes LLPS both *in vivo* and *in vitro*, and the C-terminal glycine-rich region plays a major role in GRP7 LLPS (Xu et al., 2024).

Post-translational modification such as phosphorylation can regulate LLPS (Kim et al., 2019), and various phosphorylation sites including Y^111^, S^112^, S^132^, Y^138^, S^139^, and S^140^ have been found in the IDR region of GRP7 (Xu et al., 2024). Ser^132^, which is subject to natural selection, can be phosphorylated by FER (Wang et al., 2020), implying that FER-mediated GRP7 phosphorylation at S^132^ may contribute to GRP7 LLPS and thermal fluctuations. The mutation of these phosphorylation sites, such as replacing S^132^ with non-phosphorylatable residues (Tyr-to-Ala or Ser-to-Ala mutations) in the IDR domain of GRP7, resulted in the absence of visible phase separation, indicating the importance of FER-mediated GRP7 phosphorylation for its LLPS (Xu et al., 2024). The inability to rescue the deficient heat tolerance of *grp7* mutant with non-phase-separating GRP7 variants further supports the essential role of GRP7 LLPS in responding to temperature changes (Xu et al., 2024). Additionally, GRP7 LLPS in the cytoplasm contributes to the formation of SGs containing RNA, EUKARYOTIC INITIATION FACTOR 4E1 (eIF4E1), COLD SHOCK PROTEIN 1 (CSP1), and CSP3, leading to translation inhibition, defective protein synthesis, and impaired root growth under temperature fluctuations (Xu et al., 2024). Collectively, high temperature induces RALF1 clustering (Liu et al., 2024b), and RALF1-FER module facilitates GRP7 LLPS through phosphorylation enhancing seedling temperature resilience via translational regulations (**Figure 1C**; Xu et al., 2024).

## 4 Future perspectives

In summary, these significant findings provide novel insights into RALF signaling in regulation of plant development and stress responses via LLPS (Xu et al., 2024; Liu et al., 2024b). However, open question needs to be verified in the future. It is clear that RALF peptides induce alkalization of the extracellular compartment of plant cells (Pearce et al., 2001). Hence, it is likely that the formation of pectin-RALF condensates requires an alkaline pH condition. In plants, the H^+^-ATPases (AHAs) play crucial roles in maintaining apoplast and the cell wall pH homeostasis (Tsai and Schmidt, 2021; Li et al., 2022). Further investigation is needed to determine how H^+^-ATPases contribute to the formation of these condensates. The deHG is critical for the formation of pectin-RALF condensates and RALF-triggered signaling (Moussu et al., 2023; Schoenaers et al., 2024; Liu et al., 2024b). Pectin methylesterases (PMEs) are the main enzymes responsible for deHG production (Du et al., 2022). It is suggested that RALF22 binds to LLG1 and FER to increase cell wall pH to activate PME activity, resulting in demethylesterification of HG and pectin-RALF22-LRX1/2 condensates formation (Du et al., 2022; Schoenaers et al., 2024). Several PMEs such as QUASIMODO2 (QUA2) and COTTON GOLGI-RELATED2 (CGR2) have been identified (Du et al., 2022), and their activity requires a more alkalized pH (Sénéchal et al., 2015; Schoenaers et al., 2024). The unanswered question is that how RALF peptides (in)directly regulate the PMEs activity via the H^+^-ATPases or unknown H^+^ carriers to maintain a befitting level of deHG. On the other hand, the PSY1 peptide functions antagonistically with RALF peptides to regulate cellular pH by controlling AHAs and driving H^+^ fluxes across the membrane (Fuglsang et al., 2014; Gjetting et al., 2020). It is unclear how plants equilibrate the levels of PSY1 and RALF peptides to create a befitting pH environment through AHAs or undefined H^+^ transporters or channels (Tsai and Schmidt, 2021; Li et al., 2022) to enable the formation of pectin-RALF condensates (Moussu et al., 2023; Schoenaers et al., 2024; Liu et al., 2024b). It is also unknown whether these pectin-RALF condensates are involved in ROP signaling activation. Future research is needed to understand how pectin-RALF condensate-mediated cell surface responses are translated into downstream (post)transcriptional and (post)translational regulations.

In a previous study, 452 GRP7 binding targets have been identified (Meyer et al., 2017). It is intriguing to examine whether these GRP7 interacting proteins can be incorporated to GRP7 condensates to mediate temperature fluctuations and other biological processes. Furthermore, numerous proteins interacting with FER have been discovered (Du et al., 2016; Li et al., 2018); targeting FER-interacting proteins with IDRs/LCDs could unveil new components involved in transmitting RALF-FER signals through LLPS in diverse plant developmental and adaptive processes. Notably, a temperature-sensitive site (Gly-41) in the extracellular domain of FER does not impact FER ability to perceive external cues at normal temperatures but impedes root hair formation at elevated temperatures (Kim et al., 2021). It is intriguing to test whether the Gly-41 site of FER is also important for GRP7 LLPS. FER and GRP7 also serve various functions in plant development beyond temperature responsiveness (Wang et al., 2020; Cheung, 2024; Xu et al., 2024). Further research is necessary to determine whether GRP7 LLPS contributes to additional biological processes in a RALF-FER module-dependent manner.

Taken together, LLPS plays a crucial role in RALF signaling activation. Identifying new proteins with IDRs/LCDs that can interact with RALF, RALF receptors, or pectin will aid in elucidating the mechanisms of LLPS in mediating RALF signaling and in developing innovative strategies to enhance crop growth and stress tolerance by modulating RALF pathways.

## Author contributions

ZZ: Writing – original draft. HD: Writing – original draft. SH: Writing – original draft. HH: Conceptualization, Funding acquisition, Writing – review & editing.
